# Vegetal memory through the lens of transcriptomic changes – recent progress and future practical prospects for exploiting plant transcriptional memory

**DOI:** 10.1080/15592324.2024.2383515

**Published:** 2024-07-30

**Authors:** Dóra Farkas, Judit Dobránszki

**Affiliations:** Centre for Agricultural Genomics and Biotechnology, Faculty of the Agricultural and Food Science and Environmental Management, University of Debrecen, Nyíregyháza, Hungary

**Keywords:** Non-coding RNAs, RNA interference, priming, cross-acclimation, memory modification, associative learning

## Abstract

Plant memory plays an important role in the efficient and rapid acclimation to a swiftly changing environment. In addition, since plant memory can be inherited, it is also of adaptive and evolutionary importance. The ability of a plant to store, retain, retrieve and delete information on acquired experience is based on cellular, biochemical and molecular networks in the plants. This review offers an up-to-date overview on the formation, types, checkpoints of plant memory based on our current knowledge and focusing on its transcriptional aspects, the transcriptional memory. Roles of long and small non-coding RNAs are summarized in the regulation, formation and the cooperation between the different layers of the plant memory, i.e. in the establishment of epigenetic changes associated with memory formation in plants. The RNA interference mechanisms at the RNA and DNA level and the interplays between them are also presented. Furthermore, this review gives an insight of how exploitation of plant transcriptional memory may provide new opportunities for elaborating promising cost-efficient, and effective strategies to cope with the ever-changing environmental perturbations, caused by climate change. The potentials of plant memory-based methods, such as crop priming, cross acclimatization, memory modification by miRNAs and associative use of plant memory, in the future’s agriculture are also discussed.

## 1. Introduction

Although it seems like a paradigm shift and is still the subject of intense debate, whether plants can learn,^1–6^ the first experiments on plant learning were conducted more than a century ago. Pfeffer^7^ described the change in sensitivity of *Mimosa pudica* L. plant to a repeated mechanical cue (touch), which phenomenon was called as habituation. Still in the beginning of the last century, Bose^8^ demonstrated that plants were able to discriminate between stimuli by being able to respond to mechanical stimulus produced by a finger touch when they were already habituated to mechanical stimulus produced by a drop of water. Gagliano et al.^9^ evinced that leaf-closure of *Mimosa pudica* L. ceased after repeated physical stimulation. Rapid leaf-closure of mimosa is a defense tactic against herbivores. However, when the mechanical stimulation occurred repeatedly but without herbivore attack, the plant learned not to close its leaves. This habituation was long-lasting, i.e. even after 28 days the plant remembered the acquired information. In 2017, Reed-Guy et al.^10^ studied the leaf closure time of *Mimosa pudica* L. after touching the leaves and found that in some cases, closure time increased with the number of trials suggesting that sensitization may also occur. Therefore, this result did not rule out the finding of Gagliano et al.^9^ due to the fact that the response to stimuli depends on the strength and exposure time of the stimuli. The stimuli used in these experiments, the number of touches and the exposure time were different.

Learning, if defined functionally, can be described as a lasting and adaptive change in a living organism or even artificial system resulting from interaction with the environment.^11^ Learned behavior forms if it is appropriate for flourishing and reproducing during the current environmental conditions. Learning, furthermore, assumes the presence of a memory to store the acquired information. Vegetal or plant memory refers to the ability of plants to store and retrieve information after previous exposure to stimuli thus increasing their fitness. This information-storing capacity enables plants to cope with environmental changes.^12–14^

The ideal growing circumstances of plants vary according to the species. Each environmental parameter can be described with a bell curve, which shows the optimal, sub-optimal and lethal growing conditions. Naturally, plants are able to grow under unfavorable conditions, but it may entail serious disadvantage for reproduction and yield. Any biotic (bacteria, viruses, fungi, pathogens) or abiotic (drought, salt, heat, cold, touch, wind, wounding, etc.) environmental cues and signals can become stressors. Stress may occur at various stages of growth; therefore, the impact of them may vary depending on the state of the plant. Stressors can trigger molecular events centered around epigenomic changes and including also specific transcriptomic changes and may lead to memory formation in plants. Regarding the response of a plant from stress/stimulus perception through processing to memory formation, there is a spatial organizational hierarchy, such as ecological, whole plant, cytological-biochemical and molecular including chromatin and transcriptome levels.^15^

In this review paper, we intend to focus on the transcriptomic layer of plant memory, as well as its interplay with epigenetic layer of plant memory. Furthermore, we make an attempt to present how exploitation of plant transcriptional memory may provide new opportunities in future agriculture.

## 2. Memory formation

Memory is the ability of a living organism to store, return and retrieve information based on acquired experience. Impacts on plants caused by various abiotic and biotic stimuli can lead to physiological changes that affect their growth, development and overall well-being. Time gaps between the initial stimuli and the later triggering stimuli therefore require information storage and maintenance.^14,16^ The presence of memory has been reported not only in plants but for a broad range of taxa that includes microbes,^17,18^ invertebrates^19^ and vertebrates.^20^ Memory storage for organisms that possess complex nervous systems is heavily based on specialized sites of cell–cell contact (connecting nerve cells within the nervous system), synapses and their change within.^21,22^ Several studies in other different kingdoms (including fungi – especially the yeast *Saccharomyces cerevisiae*, bacteria and plants) have been conducted to bring to light the depths of mechanisms related to priming, and memory formation. Regarding organisms without nervous systems, the current state of knowledge of the general idea concerning memory is still in its infancy.^23^ For these organisms, memory is divided into two main categories, namely external and somatic memory. External memory refers to signals deposited in the external environment (f.e. plasmodial slime molds avoid areas where previously slime has been deposited), whereas somatic memory is a result of non-genetic cell physiological and/or epigenetic changes.^24,25^

Pre-exposure to a stress/stimuli prepares the vegetation to the stimuli acting as eliciting factors that can impact (the life cycle of) the plant. An increasing number of cases show that a brief exposure to moderate, non-lethal stress can prepare the plant to later stress even with a prolonged period of no-stress in between the two events. This kind of pre-exposure plants to non-life-threatening stress is called priming, which process can enhance the individual plant’s fitness and capacity to respond to environmental stimuli faster and more efficiently.^14,26^ These occasional, mild stress events may cause stress imprint that often results in the formation of some kind of memory.^26^ There is growing evidence that plants can exhibit a form of learning based on “plant memory” (or “vegetal memory”) despite that they do not have a defined nervous system or a brain.^27^ There are a lot of synonyms used for priming in literature that have just a shade of difference. These synonyms are the following: conditioning, hardening, training, habituation, familiarization, acclimation, imprinting, sensitization, preconditioning.^27^

Acclimation, hardening, familiarization or training synonyms are used in cases when the exposure to the first stress or stimuli is recurrent. The continuous stress changes the plant's response intensity to environmental stimuli. In case of reducing the strength of response, therefore learning to ignore harmless stimulus, terms of familiarization and habituation are used.^15,28^

In some cases, a decrease in responsiveness can be attributed to effector fatigue or sensory adaptation, so to rule out these alternative explanations some tests may be necessary. Effector mechanisms are vital for ensuring the proper functioning of response expressions. Effector fatigue occurs if these functions cannot work properly (f.e. cavitation fatigue in sunflowers, due to the response systems depletion).^29^ In case of ruling out fatigue, a procedure called “dishabituation” is used, where plants receive a secondary stimulus that triggers the target response. If after the treatment with this secondary stimulus and then with the original stimulus again shows the same results, effector fatigue can be excluded.^30^ In other cases, where as a result of sustained stress exposure, the sensory organs change, it is called sensory adaptation. Sensory adaptation refers to the phenomena where after exposure to a stimulus a reduction in sensitivity occurs (f.e. after exposure to low levels of auxin, corn coleoptile segment’s increase in responsiveness wasn’t parallel to the rate of uptake of the hormone).^31^

If the response intensity rises after continuous exposure to stimuli, alternative terms are used such as sensitization. In case of sensitization, after an unpleasant, painful stimulus, the plants may show increasingly violent responses.^28^ Sensitization is an important immunological phenomenon that may occur during symbiosis, parasitism and recovery of diseases, and as a reaction to antibodies and foreign bodies within the host plant. After sensitization, acquired immunity may arise against the before mentioned events.^32^

**Figure 1.**
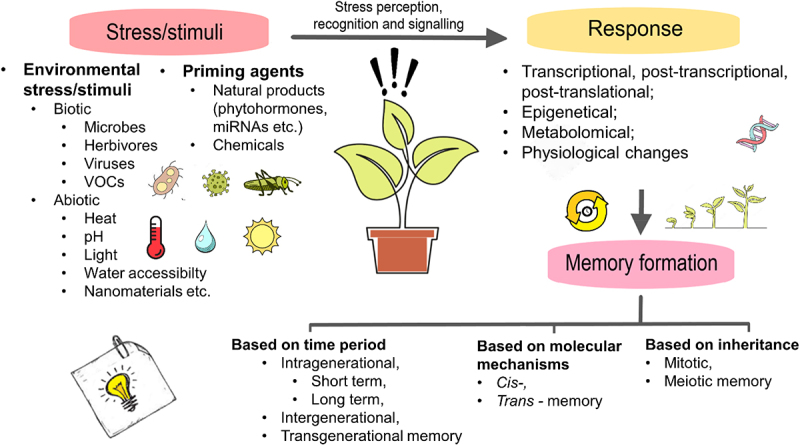
Main processes from stimulus to memory in plants.

A stimulus can result in four major types of changes in the plant: physiological, transcriptional, metabolic and epigenetic changes.^33^ Although most of these changes disappear in the absence of the initial stimuli, some changes may last, resulting in memory formation (Figure 1). Physiological changes include, for example, phytohormone level fluctuation (f.e. elevated expression of abscisic acid (ABA), jasmonic acid (JA) biosynthesis and other abiotic stress-related genes after treatment with salicylic acid or cold priming followed by mild freezing stress).^34^ The transcription factors, post-transcriptional modifications (altered, sustained) are grouped within transcriptional changes caused by priming. Metabolic changes include f.e. Ca^2+^ and ROS level shifts.^35^ Another grouping option to stress memorization is based on molecular mechanisms. According to this categorization, epigenetic processes (DNA methylation and histone post-translational modifications), modifications of regulatory proteins and accumulation of latent signaling compounds (molecules, key metabolites) create plant memory, with the possibility of transmission to their offspring. The duration of these changes mainly depends on the nature and the duration of the priming stress, and the underlying mechanisms.^12,13,36^

Not only genetic but also epigenetic mechanisms play a role in the formation of the phenotype, and the result of their cooperation is phenotypic variance, which enables plants to modify their growth behavior in response to environmental changes.^37^

To better understand how an organism can learn without a defined nervous system, or a brain, biologists turn to the ‘omics’ for answers. As a consequence of the advancement of omics technologies, we could gather physiological, epigenomic and transcriptomic evidences that can provide insights into the plant stress memory.^36,38^

As for molecular mechanisms, the best characterized mechanism is a modified transcriptional response which can be a result of epigenetic processes (DNA methylation pattern changes, histone post-translational modifications), regulatory protein level shift, accumulation of signaling compounds (molecules, metabolites) and transcriptional feedback loops.^39^

The level of gene expression depends on the nature of the DNA nucleotide sequence (genome) and its epigenetic state (epigenome, epigenetic memory). The epigenome is a collective name that includes all epigenetic marks on the chromatin, that is the result of chemical changes on DNA, which do not change the base order of the DNA but alternate the transcription of genes.^40^ The state of the epigenome determines gene silencing and activation by altering the eu- and heterochromatin states. In the euchromatin arrangement, the chromosome has a looser structure, the DNA is not tightly bound to the histone proteins. In this arrangement, gene expression can be active if the appropriate transcriptional factors are present, since the transcription machinery has access to the DNA. In contrast, heterochromatin is more compact and generally less accessible to cellular and transcriptional machinery, gene expression does not occur from chromatin with this structure, therefore genes are silenced. The rearrangement of the chromatin structure can take place in several ways.^41^ Epigenetic memory consists of chromatin remodeling, histone modifications, DNA methylation, nucleosome partitioning and non-coding RNA-mediated regulation generating a highly complex system.^36,42–44^ Based on recent “omics” research, the blue print of the plant memory is in the epigenome.^45^ Its modification acts through alterations in the gene expression resulting in metabolic and physiological alterations that are responsible for increasing the plant fitness. The existing epigenetic patterns with distinct distribution and level of methylation, acetylation and other epigenetic marks can form epialleles, which are stably carried on to the future generations.^46^

## 3. Memory types

Vegetal memory types can be grouped in several ways, depending on inheritance, duration of gene activation or silencing, and molecular mechanisms. Based on inheritance, there are mitotic and meiotic memory categories.^39^ Mitotic memory subsists through clonal reproduction and mitotic cell divisions. In some cases, it can be equivalent to somatic or intragenerational memory, because it affects mainly the current generation of the species. This type of memory, which may persist through an individual plant’s lifespan, is stress-induced and transcriptional-based.^36,47,48^ Plant seed genomic imprinting or priming, where depending on whether the gene was inherited through a male or a female gamete, leads to differential allelic expression. In plants, this phenomenon occurs in embryo-nourishing tissues (endosperms). In the endosperm of maize, *MEF1* and *FIE2* genes were differently methylated, but not in gametes, suggesting that the establishment of methylation patterns happens after fertilization, through mitotic cell divisions.^49,50^

Meiotic memory can influence future sexual generations as well, for the reason that it subsists through meiotic cell divisions.^36^ This memory can last within the next few progenies, without exposing the offspring to the stress again.^47^ Interestingly, pathogenic flagellin peptide or UV-C treated *Arabidopsis* plants show increased levels of homologous recombination.^51,52^ The untreated progeny of the phenotype possessing elevated levels of homologous recombination showed the same traits across four or more generations. This result suggests that epigenetic memory acts as a dominant trait, which can become meiotically (either maternally or paternally) heritable to successive generations.^53^

Based on the length of the time-period until the memory remains intact, there are three main classes of memory: intra-, inter- and transgenerational memory. Intragenerational memory affects the individual plant and occurs during the span of one generation. If the memory recall lasts only within a limited period of time, it is called short-term memory (usually about some days to few weeks). However, in case of long-term memory, the stored information can persist across multiple developmental stages within the current life cycle.^36,48^ Intergenerational memory extends to the next generation, while transgenerational memory subsists through the next two or more generations without stressors/stimuli. There is more and more evidence that shows that the separation of inter- and transgenerational memory is necessary due to the fact that the memory of a stimulus may fade across several progenies.^54^

Despite the numerous studies pointing out the transgenerational inheritance of priming, relatively few studies deal with the inheritance stability of epigenetic changes. Therefore, the focus of new research papers should be on what regulatory elements can enable epigenetic stability throughout the next generations, and what contributes to the inheritance of the gene expression patterns. Also investigating the inter- and transgenerational memory of plants, the maternal (or paternal) factor should be taken into account, especially when the immediate progeny develops on or near the mother plant.^55,56^

## 4. Transcriptional memory

A possible manifestation of memories is a modified transcriptional response. Functional requirements for transcriptional memory are at the transcript levels. Following the initial stimuli, transcripts are produced from responsive genes at a certain level. The recovery period will last until all the metabolite, protein and transcriptional levels return to the normal state (baseline, pre-stress state). After the recovery phase, if the plant receives a stimulus and shows significantly different transcriptional levels of the same genes, even though the level and duration of the stresses are identical, it meets the criteria for transcriptional memory.^57^

Using repetitive dehydration/rehydration system on wild type and *atx1 Arabidopsis* plants (*Arabidopsis thaliana)*, Ding et al.^58^ analyzed four dehydration-inducible genes (*RD29A, RD29B, RAB18, COR15A*) as markers of stress memory. Two of the four genes were unaffected by repeated stress exposure (non-trainable genes, *RD29A, COR15A*), while the other two (*RD29B, RAB18*) showed increased levels of transcription. Studying the temporal aspects of memory, they discovered that in the plants that were previously exposed to inducing signals, and then normally watered for 3, 5, and 7 days, the above-mentioned transcriptional memory was maintained only for 5 days. However, it was deleted after 7 days. When the plants were stressed again, then 7 days of watering, the transcript levels changed from increased to “normal” levels (similar to the levels observed after the first exposure to dehydration).^57^

**Figure 2.**
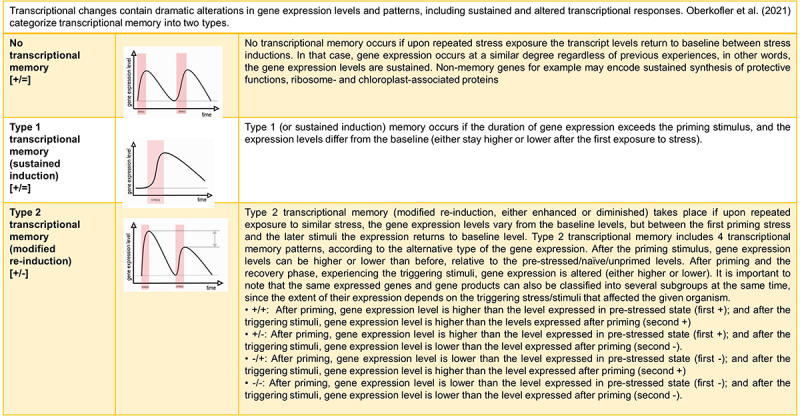
Transcriptional memory types 59,60.

Figure 2 depicts the different types of transcriptional memory.^59,60^ No transcriptional memory happens if gene expression occurs at a similar degree upon repeated stress exposure. Transcriptional memory is the phrase used, when gene expression occurs in a different degree (either sustained levels of expressed genes or modified re-induction of genes) upon the same circumstances.

### 4.1. Cis and trans memory

Based on the molecular mechanisms, the main two memory categories can be defined as *cis* and *trans* memory.

*Cis* memory information is physically located in the chromatin itself, which includes DNA methylation and histone post-translational modifications. This type of memory has a key role in the vernalization process. Berry et al.^61^ described the *cis*-memory storage capability of *FLOWERING LOCUS C* (*FLC)* chromatin by adding two different tags/reporters to two copies of *FLC* genes within the cells of *Arabidopsis* plants. This allowed the experimenters to track each copy of the gene in individual cells, and they found out that it is possible, that one copy remains switched on, while the other is turned off at the same time. This proves, that the memory of cold is not stored globally (because in that case, the two copies would show the same gene activity), rather, in the local chromatin structure. In that case, the histone modifications cause chromatin structure alterations. The spreading of H3K27me3 histone modification silences the *FLC* epigenetically after cold stress, during recovery. Moreover, the daughter cells receive the same pattern of gene activity, after the original cell divides, hence this form of *cis* memory is heritable.^62^ Also, the vernalization process shows that memory formation can occur upon an exogenous cue in a stress-independent context.

*Trans* memory uses trans-acting feedback loops. It is mainly sustained through the concentration of a diffusible signal, which is transmitted by the cytosol partitioning, and maintained by feedback loops. There are two proposed *trans* memory mechanisms, one is the accumulation of signaling compounds (f.e. ABA) and the other is the accumulation of transcription factors.^36,38,61^ Recently, a heritable regulatory feedback-loop in *Arabidopsis thaliana* has been characterized.^54^ *REF6-HSFA2 (RELATIVE EARLY FLOWERING 6 - HEAT-SHOCK TRANSCRIPTION FACTOR A2)* positive feedback loop operates in the transgenerational adaptation after heat stress (HS). *REF6* and *HSFA2* mutually assist each other’s transcription. By continuously activating each other’s expression, as the cell grows and divides, there will be a constant concentration of these two components. Therefore, maintaining the active state of *HSFA2*, this feedback-loop creates epigenetic memory, which can be heritable, and therefore promotes transgenerational memory formation.

### 4.2. Pathways related to the link between epigenetic and transcriptional memory layers

Several epigenetic changes underlying and related to transcriptional memory have been discovered in recent decades. The changes affecting the transcription (either activating or silencing it) arise mostly from chemical, covalent modifications of chromatin that alter both DNA and histone proteins. At the transcriptional and post-transcriptional levels, non-coding RNAs regulate gene expression in a sequence-specific manner by influencing the distribution patterns of epigenetic marks.^45,63^

**Figure 3.**
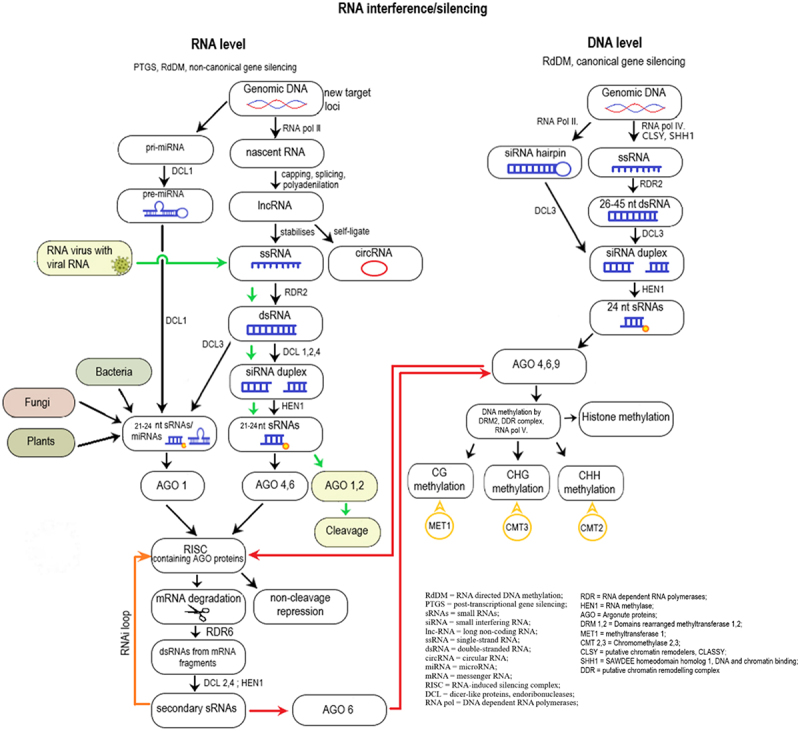
A summary of RNA interference/silencing mechanisms at the RNA and DNA level.

Figure 3 summarizes the pathways that relate to the different layers of plant memory including the interplay and cooperation between epigenetic and transcriptional memory. The relation of DNA methylation, gene expression changes and the regulatory role of non-coding regions is still poorly explained. The main pathways related to plant memory establishment and maintenance are post-transcriptional gene silencing (PTGS), RNA-directed DNA methylation (RdDM, canonical and non-canonical), the RNAi loop, DNA and histone modifications.

Plants attain targeted DNA methylation through RNA-directed DNA methylation (RdDM). RdDM plays an important role in *de novo* DNA methylation and maintenance in all sequence contexts, and in case of foreign DNA in the initiation of transcriptional silencing. The RdDM process involves RNA molecules such as small interfering RNA (siRNA) or long non-coding RNA (lncRNA) to guide enzymes to add methyl groups to specific DNA regions. A series of methyl-binding proteins can attach to the methylated cytosine, modifying the chromatin structure, which prevent the transcription machinery from accessing the DNA. Therefore, RdDM also regulates (suppresses) transposable element (TE) activity that can be heritable due to the self-reinforcing nature of RdDM.^64,65^

The heritable nature of DNA methylation pattern alterations suggests that the process acts as a form of memory, therefore allowing the plant to respond more efficiently to future stress events.^66^ Functional sRNA biogenesis pathways play a major role in the resistance against insect attack via DNA methylation changes that can be transmitted to descendants.^67,68^ Boyko et al.^69^ reported that salt or heat stress-induced DNA methylation changes required several RdDM-related proteins, suggesting that RdDM is vital for the maintenance of stress-related DNA methylation patterns. They stated, that even in the absence of the original stimuli, the memory of stress can be heritable in *Arabidopsis* plants. The silencing nature of RdDM also serves as a form of memory, due to the fact, that after a prior infection, genome-integrated TEs or viruses remain silenced. Moreover, these integrated sequences act as a formula to combat against future invasions by similar agents. Consequently, plant epigenome can potentially be altered and maintained by RdDM, providing the possibility to modulate future stress responses in the current and future generations as well.

RdDM depends on the collaboration of DNA-dependent RNA polymerases (RNA Pol), RNA-dependent RNA polymerases (RDR), dicer-like proteins (endoribonucleases, DCL), RNA methylase (HEN1), and argonaute proteins (AGO). The establishment of DNA methylation is achieved through the recruitment of domains rearranged methyltransferase 1,2 (DRM1,2) and maintained by methyltransferase1 (MET1) or chromomethylase2, 3 (CMT2, CMT3).^70^ RdDM can take place in a canonical and non-canonical manner, where the main difference is the origin and biogenesis of sRNAs involved (Figure 3).

The canonical RdDM pathway, where long-term maintenance of DNA silencing and repressive histone modifications happen, can be divided into two main processes: 1) the biogenesis of small RNAs (sRNAs), 2) DNA methylation of target loci and histone modifications. This process reinforces existing DNA methylation patterns by positive feedback-loops.^71^ During sRNA production, RNA Pol IV is recruited by CLASSY (putative chromatin remodelers, CLSY) and SAWDEE homeodomain homolog 1 (DNA and chromatin binding, SHH1) to the target loci that need to be silenced, where transcription of ssRNAs begins. Then, RDR2 creates a second strand of RNA, forming dsRNA, which step is followed by the DCL3 cleavage, resulting in sRNAs. Nearly all sRNAs are produced by the cooperation of RNA Pol IV, RDR2 and DCL3 (except in case of hairpin structures, where only RNA Pol II and DCL3 are needed). One strand from sRNA is loaded into AGO proteins (AGO 4,6 or 9), forming a protein-RNA duplex. This duplex can locate and binds to a complementary RNA sequence along an RNA scaffold (that was previously formed by the plant-specific RNA Pol V). This structure recruits DRM2, with the help of other complexes (Suppressor of Ty insertion 5-like, involved in *de novo* 2-IDN2 paralog complex and RNA Pol V subunit NRPE1), which methylate nearby DNA structures (Figure 3).

Non-canonical RdDM compared to its canonical counterpart targets relatively few loci while establishing initial *de novo* DNA methylation and post-transcriptional gene silencing (PTGS). The pathway begins with sRNA biogenesis (similar to the canonical way). The first step is nascent RNA transcription from genomic DNA (intergenic regions, TE insertions, introns, exons, transposons, repetitive sequences, coding regions, NATs, viruses) via RNA polymerases (RNA Pol II, III, IV, V) followed by capping/splicing/polyadenylation that results in lncRNAs (long non-coding RNA, mi or siRNA precursor).^72^ These lncRNAs may self-ligate forming circular RNAs, or stabilize, generating single-strand RNAs (ssRNAs). From ssRNAs, the RDR2 enzyme forms double stranded RNAs (dsRNAs), then an endoribonuclease (DCL1,2,3,4) cleaves them to smaller pieces (si/miRNA duplexes). After that, HEN1 RNA methylase adds a methyl group to them, forming 21–22 nt sRNAs, miRNAs. These sRNAs bind to AGO proteins (forming protein-sRNA duplex), then get loaded into the RNA-induced silencing complex (RISC). RISC is a fundamental element of RNA interference. RNA interference (RNAi) is a cellular process, where special small, non-coding RNAs [sRNAs, e.g. small interfering RNA (siRNA), micro RNA (miRNA)] bind to complementary RNA sequences, thereby it inhibits the protein production from those target RNAs via non-cleavage repression. RNAi is also accountable of this sequence-specific post-transcriptional gene silencing (PTGS) which means that non-canonical RdDM may be a byproduct of PTGS pathways. The central components of RISC are AGO proteins bounded with sRNAs that cause cleavage in the target mRNA. Thereby it generates abnormal sequences which the organism does not recognize and therefore breaks down. In simple terms, RNAi inhibits gene expression by degrading mRNAs or blocs the translation of the target mRNA molecules. By degrading the mRNA, the organism generates new fragments that can act as secondary sRNAs creating a positive feedback-loop. Once the initial silent state is established, RNA Pol IV and CLSY and SHH1 proteins are recruited to the locus and canonical gene silencing takes place^71^ (Figure 3).

While both RNAi (non-canonical RdDM) and canonical RdDM involve RNA molecules and contribute to gene regulation, they operate at different levels: RNAi primarily affects RNA molecules, whereas canonical RdDM modifies DNA through the guidance of RNA molecules^73^ (Figure 3).

Pre-existing DNA methylation at symmetric (CG) and semi-symmetric (CHG) sequence contexts can be maintained immediately after the S-phase at the semi-conservative replication of DNA. The DNA methyltransferase enzymes copy the methylation pattern to the newly synthetized unmethylated strand in an sRNA independent manner. These enzymes are the plant specific CMT3 and MET1. Through successive rounds of DNA replications, these patterns subsist from mother to daughter cells enabling the plant to inherit these changes. However, in case of asymmetric (CHH) methylation patterns, the semi-conservative replication of methylation is theoretically impossible. Therefore, continuous *de novo* DNA methylation activities, which are sRNA dependent, are essential in reserving the pattern. Therefore, this process is a self-reinforcing loop^74^ (Figure 3).

### 4.3. The role of non-coding RNAs (ncRNAs) in plant memory

The intensity of the modified transcriptional response and type of response mechanism changes, depending on how much time has passed from the initial stimuli. The plants first feedback to environmental challenge is mainly intensive ROS formation followed by callose accumulation. These early-late responses may transform into enduring responses (memories), such as unphosphorylated MAPK accumulation, histone modification, DNA methylation and nucleosome or chromatin remodeling. In the long run, the aforementioned changes may be inherited to the offspring.^75^

More and more evidence has been accumulated regarding the role of different types of non-coding RNAs in the establishment and generation of epigenetic changes associated with memory formation in plants. Transcription, RNA editing, splicing, translation and turnover, epigenetic memory and chromatin remodeling are regulated by ncRNAs, despite the fact that they are unable to undergo protein translation. Furthermore, they are essential for managing genomic stability (by protecting the genome from both external and internal factors) and plant development (including growth, immunity to different abiotic and biotic stressors, senescence).^76–78^ Target genes are regulated by ncRNAs either in an antagonistic or synergistic way, for the fact that the regulatory pathways maintained by ncRNAs are interconnected^79^ (Figure 3).

There are two main categories of non-coding RNAs: long (lncRNAs) and small non-coding RNAs (sncRNAs).^80,81^ Both lncRNAs and sncRNAs share regulatory targets, for instance, protein-coding genes or genomic loci; therefore, their interactions and connections modulate gene expression together. These RNAs have a role in epigenetic modifications, for example, some lncRNAS can guide chromatin-modifying complexes to specific genomic regions, and similarly, sncRNAs can also be involved in the establishment and maintenance of epigenetic marks. Although these RNAs have distinct functions, there can be functional overlaps in certain contexts. In particular, some lncRNAs can compete like a decoy with miRNAs (a sub-group of sncRNAs, microRNAs) for target mRNA binding or can interact with the miRNA machinery^82^ (Figure 3).

Long non-coding RNAs (lncRNAs) that lack polypeptide potential and bigger than 200 nt are classified into five different groups.^80,81^ Long-intergenic ncRNAs (lincRNAs), or in other words macroRNAs or long intervening ncRNAs, are weakly spliced and show tissue-specific expression. They have a trans-regulating role with a rapid turnover rate. LincRNAs lie in gene deserts, meaning a minimum of 5 kb away from protein-coding regions.^82,83^ This group consists of five different sub-groups, according to their association with specific regions, namely upstream antisense RNAs (uaRNAs), promoter-associated long RNAs (PALRs), telomeric repeat-containing RNAs (TERRAs), enhancer RNAs (eRNAs) and promoter upstream transcripts (PROMPTs), in which the latter two are short-lived and mainly found in humans.^84^

Transposable element-derived lncRNAs (TE-lncRNAs) are generated from TEs (jumping or copy/cut-paste genes) and act as precursors to micro RNAs (miRNAs) and small interfering RNAs (siRNAs). Based on the generating mechanism, there are Class I (RNA-mediated, reverse-transcription based copy and paste) and Class II (DNA element based, cut and paste) TE-lncRNAs.^83,85,86^

Intron-derived lncRNAs (incRNAs) contain poly(A) modifications; therefore, they are highly conserved and stable. These incRNAs arise from introns that are within protein-coding genes. Based on their origin, there are totally intronic RNAs (TINs) and partially intronic RNAs (PINs) which transcripts are regulated by various transcriptional pathways.^87^

Natural antisense transcripts (NATs) have a role in gene expression regulation (silencing) through RNA-RNA and RNA-DNA interactions. *Cis* NATs originate from opposite strands of coding DNA (which is therefore non-coding), making it a complementary sequence to mRNAs. By forming RNA duplexes with the corresponding mRNA, they silence gene expression. NATs can regulate alternative splicing by interacting with pre-mRNA, therefore generating mRNA isoforms, and also participate in RNA editing (insertion, deletion, substitution of nucleotides). Enhancer/suppressor NATs interact with the regulatory regions of target genes. Trans NATs trigger the production of specific siRNAs, and they are transcribed from a different genomic locus than the target gene. They also can possess a complementary sequence of the target gene’s mRNA.^88^

Circular RNAs (circRNAs) are covalently closed stable and highly conserved. They originate from pre-mRNAs by back-splicing of exons or from the cytoplasm. They function as endogenous target mimics of miRNAs, miRNA sponges or protein scaffolds/templates.^89^

From a functional perspective, there are two kinds of lncRNAs: *cis* and *trans* acting lncRNAs. *Cis*-lncRNAs are accountable of transcriptional interference and chromatin modifications. These molecules can interact with transcriptional factors and cause the blockage of preinitiation complex formation by promoter binding. *Cis*-lncRNAs participate in the recruitment or decoy of chromatin remodeling complexes and transcriptional machinery. On the contrary, *trans*-lncRNAs are responsible of binding to transcription elongation factors, chromatin modifying complexes, RNA polymerases or ribosomes which affect transcription. They target distant gene loci, and they are able to function independently of target sequence complementarity. They participate in post-translational regulation when directly binding to or modulating mRNA splicing factors or hybridizing with mRNA, they block splicing.^90^

LncRNAs can be processed into small ncRNAs (sncRNAs), consequently they serve as precursors or scaffolds for sncRNA biogenesis, and also lncRNAs may guide the processing and maturation of sncRNAs. Small ncRNAs include micro-RNAS (miRNAs) and small interfering RNAs (siRNAs), which are key regulators in transcriptional and post-transcriptional gene regulation. The source of siRNAs can be endogenous (TEs, repetitive elements or centromeres) or exogenous (viruses, aberrant inverted repeats). Another type of classification is based on the origin of sncRNAs, where we differentiate between trans-acting siRNAs (tasiRNAs), natural-antisense siRNAs (nat-siRNAs), repeat-associated siRNAs (rasiRNAs), heterochromatic siRNAs (hc-siRNAs) and viral siRNAs (vi-siRNAS).^90^

## 5. Reset, checkpoints

Sometimes it is advantageous for the plants to remember past events and store the information gathered from the past experience for later use. Other times it could hinder the recovery process or slow down the development of the plant. In that case, it could be beneficial to forget or delete the once acquired information to maximize the growth under favorable conditions.^23,91^ Therefore, the question arises: how long should a memory of an environmental cue last? To reset the chromatin states, some kind of “checkpoints” are needed, whether to decide if a memory should be maintained or should be deleted.^66,92^ Iwasaki and Paszkowski^63^ proposed a possible mechanism to remedy the chromatin state, that is similar to cell cycle checkpoints, but instead of DNA damage detection and repair, these checkpoints ensure epigenetic memory deletion. Their work demonstrated the requirement of *Decrease in DNA Methylation 1* (DDM1) and *Morpheus’ Molecule 1* (MOM1) proteins in perpetuating certain epigenetic stages (for example, to prevent transgenerational memory formation).

There are a few proposed mechanisms about the deletion of other kinds of memories (or resetting), based on the storage method. The memories stored in the form of proteins are usually erased by protein degradation, which can be achieved through several different systems.^14,93^

The information stored in the form of sustained expression of RNAs (e.g. micro RNAs) or the accumulation of messenger RNAs may ensure memory formation. The RNA metabolism is a key regulatory point to reset (clear all the transcriptomes responsive to the stress) or selectively stabilize RNA-based memory formation in plants. Exonuclease decay, miRNA/siRNA silencing, post-transcriptional gene silencing (PTGS) or RdDM pathways may cause RNA degradation, therefore inhibiting the initiation of epigenetic memories. The RNA turnover process competes with overriding strategies against the epigenetic memory mechanisms due to the fact that the same RNA substrates are used by decay and gene silencing pathways (PTGS, RdDM).^91,94^

The information stored in histone modifications may also undergo reset at developmental phase transitions. H3K27me3 establishment and maintenance facilitated at DNA replication by H3.1,^95^ at the floral repressor *FLOWERING LOCUS (FLC)* is essential to initiate flowering in *Arabidopsis thaliana*. Before or during early plant embryogenesis H3K27me3 mediated silencing may reset to assure transcriptional re-activation. The precise timing and mechanisms of this process are still not clarified.^96^

The maintenance of the pattern of intraindividual (intergenerational) epigenetic marks requires information storage through plant development and cell division, but in some instances, to prevent inheritance between generations, reset may occur. Unfortunately, the precise mechanisms underlying the process of distinguishing between the two outcomes (whether a memory should last or perish) are not clear.^97^

## 6. Future utilization of plant memory potential

### 6.1. Crop priming

There are a lot of existing strategies focused on crop improvement that are either obsolete or time-consuming (e.g. conventional breeding) or dependent on species or dosage (e.g. usage of phytohormones exogenously).^98^

As mentioned before, pre-exposing plants to mild, non-lethal stress (that is stress priming) can induce the formation of stress memory, which result in the emergence of stress-tolerant plants in the current and future generations as well. The resulting epigenetic variation if maintained over many generation causes genetic assimilation. These kinds of changes ensure enhanced adaptation and increased expression of favorable traits (e.g. drought, heat tolerance or pest resistance).^36,99^ Theoretically, the minimal investment of resources used in the process of priming can result in improved future plant survival rate and therefore in future stress events in response to triggers may cost less all-in-all.^23^

In cold primed *Arabidopsis* plants, transcriptome analysis revealed that the modification of signal sending and transduction pathways, lipid and protein composition and gene expression pattern changes can prepare the vegetation of a subsequent stress event.^100^ Another survival strategies are the accumulation of osmolytes (sucrose, proline) that contribute to the cell membrane stabilization and enable cryoprotection, and ROS scavenging enzyme activity enhancement (SOD, APX, GR), which ensures the protection of the photosynthetic apparatus.^101,102^

Beneath the plants optimum temperature, cold stress may depress plant growth and metabolism, resulting in smaller and less productive crops.^103^ Cold priming is divided into two groups: chilling and frost/freezing stress. There are numerous cases that show that priming by cold stress can enhance the tolerance of crop plants. Due to their evolutionary path, tropical and subtropical crops (potato, tomato, bean, soybean, rice, maize, cotton) are more susceptible to chilling damage. However, plants from temperate climates (barley, oats, wheat), where they have previous experience with colder temperatures, generally are chilling tolerant but vary in frost sensitivity.^104^

Beyond the optimum range, heat stress could cause irreversible cellular damage that prompts water-use efficiency depletion, photosynthetic capacity, floral viability, yield reduction, etc. It is a well-known fact that heat priming induces heat-shock protein (HSP) expression; therefore, increased levels of these macromolecules are associated with thermo-tolerance.^105^

Singh et al.^106^ found that in *Arabidopsis thaliana (Columbia-0)* recurrent heat stress (44°C for 2 h/day for 7 days) impacts the expression of heat-stress memory genes (*APX2* and *HSP22*) by a regulator called constitutive photomorphogenesis 5A (CSN5A). CSN5A is also necessary for the deposition of H3K4me3. Based on the fact that the functionality and structure of CSN are similar to 19S proteasome;^107^ therefore, it may have a role in histone methylation and therefore in transcriptional memory in a deneddylation-independent manner.

Seed ultrasonication had an after-effect and caused increased growth of shoots and roots of 7-week-old seedlings of winter wheat (*T. aestivum* L. cv. SE15). Studying differentially expressed genes (DEGs) and differentially methylated regions and genes (DMRs, DMGs) Hidvégi et al.^108^ found a close correlation between the mRNA transcription and DNA-methylation changes. Ultrasonication was a priming technique that promoted enhanced seedling growth while modifying the methylation and transcription of several genes. This discovery is another key to understand short-term (7 days after germination) intragenerational memory of plants.

### 6.2. Cross-acclimation – a possibility for practical use of transcriptional memory

Cross-acclimation in a phenomenon that occurs when previous exposure to a certain non-lethal stress results in another stress tolerance by improving the fitness of the individuals (or even their progeny).^36,109^ The process mainly relies on the shared synergistic signaling pathways across stresses that differ in nature and vigor.^36^ This kind of priming can be advantageous for the types of stresses that mainly occur at the same time (heat-drought).^110^ If the priming stimuli and the stresses are the same, *cis*-priming/stress tolerance occurs. On the contrary, if the priming stimuli/agent and the later occurring stresses are different from one-another *trans*-priming or cross tolerance/acclimation is the adequate term used.^111^

A great example of crop cross-priming is based on spring wheat. Wang et al.^112^ exposed plants to moderate water deficit (drought stress) during the vegetative stage of growth. This prompted elevated heat and drought tolerance during grain filling and therefore caused yield loss reduction. During drought priming, the maximum electron transport rate decreased, which resulted in lower photosynthetic activity. Primed plants showed increased ABA concentration, lower energy dissipation rate and facilitated photosynthesis by increasing the carboxylation rate during grain filling.

Despite the fact that model plants are the most common subjects of research, it would also be worthwhile from an agricultural point of view to investigate the priming phenomenon extensively. An extended period (for 7 days) of mechanical stress primed 10-day-old *Arabidopsis* seedlings against the attack of necrophytic pathogens (*Alternaria brassicicola* and *Botrytis cinerea*). This priming effect was connected with cell wall modifications, accumulation of defense compounds, induction of jasmonic acid signaling and alterations in the expression of defense-related genes^113^ (Table 1).

**Table 1.** Application possibilities for cross-tolerance in model plants and crops.

Species	Applied stress parameters for cross-acclimation	Changed parameters	Type of tolerance generated		References
			Tolerance	Cross-tolerance	
*Arabidopsis thaliana* (L) Heyhn.	Priming: β-aminobutyric acid (BABA) solution applied as soil drench. Treatment: salt and drought	Provokes stomatal closure, increases drought tolerance	chemical	salinity, drought	114
Tomato (*Solanum lycopersicum* L. cv. Castlemart)	Priming: mechanical wounding 24 h prior to treatment. Treatment: 200 mM NaCL solution every 24 h	Increased protein prosystemin and jasmonic acid levels, induction Pin2 expression. Increased calmodulin-like activities (possibly LeCDPK1).	wounding	salinity	115
Rice (*Oryza sativa* L. cv. TN1)	Priming: 45°C heat shock for 1, 2 or 3 h in the dark. Treatment: CdCl_2_ 0,5 mM, 2 days	Increased H_2_O_2_ level, APX, GR activities, increased MDA content and reduced chlorosis.	heat	heavy metal	116
Pea (*Pisum sativum* L.)	Priming: 6°C for 16 weeks. Treatment: 10 mM CuSO_4_ or 5 mM CdCl_2_	Reduced PSII prohibition. Higher GSH and AsA accumulation	cold	heavy metal	117
Wheat (Tritium aestivum *L.*)	Priming: caryopses were primed with choline chloride 5 mM for 24 h, germination on sand with MHS (10d) Treatment: 150 mM NaCl for 3 weeks	Decreased leaf lipid peroxidation, increased K^+^ and Ca^2+^ in the shoot and root	oxidative	salinity	118
Austrian or Black pine (*Pinus nigra* J.F. Arnold)	Priming: drought in warmer, drier climates. Treatment: freezing climate	During drought epicuticular wax alkane levels increased, lipid composition changed, which happens to correlate to cold tolerance.	drought	cold	119
Mustard (*Brassica campestris* L)	Priming: heat shock 45°C for 5,5 h in the dark 6 h recovery at 25°C. Treatment: salt stress 150 mM NaCl, drought stress (20% PE-6000) for 48 h	Enhanced activities of APX, DHAR, GR, GST, GPX, CAT, Gly I, and Gly II. Additionally, these seedlings maintain lower levels of GSSG, H_2_O_2_, and MDA, particularly when facing salt and drought stress.	heat	salinity, drought	120
Mustard (*Brassica campestris* L)	Priming: cold shock 6°C for 5,5 h in the dark 6 h recovery at 25°C. Treatment: salt stress 150 mM NaCl, drought stress (20% PE-6000) for 48 h	Increased AsA and GSH levels, as well as a higher GSH/GSSG ratio. Enhanced activities of APX, DHAR, GR, GST, GPX, CAT, Gly I, and Gly II, while maintaining lower levels of GSSG, H_2_O_2_, and MDA.	cold	salinity, drought	121
Barley (*Hordeum vulgare* L. cv. Tokak 157/37.)	Priming: short (30 min) heat shock (HS, max 45 °C) on plants. Treatment: grown under salinity stress (SS), 200 mM NaCl for 10 days	Moderate heat stimuated plant growth and increased osmotic potential in leaves. Under heat, salt and combined stress, gene expression changed (HvAPX, Cu/Zn-SOD, HvGST, HSP17, HSP90, HSP18, HSP70, HvMT2, HvDRF1, HvMT2, BAS1).	heat	salinity	122
Soybean (Glycine max (L.) Merr.)	Priming: Static magnetic field, 200 mT for 1 h. Treatment: 0, 25, 50, 75, 100 mM NaCl for 3 days	Decreased leaf area-reduction, decreased H_2_O_2_, SOD, APX GR and POD activities, increased total AsA contents	magneto	salinity	123
Sweet pepper (*Capsicum Annum* L.)	Priming: PEG-4000, −0.2 MPa, 12 h, Treatment: 60 to 80 mM NaCl	Increased total proline content, SOD, POD, APOX activity	drought	salinity	124
*Arabidopsis thaliana* (L.) Heyhn.	Priming: mechanical stress using a soft brush (8 h intervals, 10 s twice daily) Treatment: pathogen infection	High expression of *TCH3*, *TCH4* (*XTH22*), *GSL6*, *PRX71, GST1* genes, upregulated JA and reduced SA levels, high lignin and callose deposition, decreased petiole length	mechanical	pathogens	113
Chickpea (*Cicer arietinum* L varieties PBG1 and PBG5)	Priming: mild drought for 3 days, 6-day recovery, Treatment: 12 h of different levels of heat-stress (30°C, 32°C, 34°C, and 36 °C)	Increased activity of SOD, CAT and APOX up to 34°C, decline at higher temperatures. Changes in the expression level of sHSPs	drought	heat	125
Tobacco (*Nicotiana tabacum* L.)	Priming: 14 days of 1.75 kJ m−2 day−1 4 h/day UV-B radiation and 0.2 mM H_2_O_2_, individually and combined. Treatment: 7-day drought	Increased total antioxidant capacity and foliar epidermal flavonol content, increased the activity of CAT, phenylalanine ammonia lyase and POX activities	UV, oxidative	drought	126
Olive cv. Chétoui	Priming: salinity for 21 days (200 mM), 2 months of recovery. Treatment: withdraw of water for 30 days	Accumulation of osmoticums (total sugar and proline content), improved ROS scavenging system (increase in SOD, CAT and GP activities, phenols), and increase in leaf density, raise of structural lipid content.	salinity	drought	109

APOX = ascorbate peroxidase, AsA = ascorbic acid, CAT = catalase, Cu/Zn-SOD = copper-zinc superoxide dismutase, DHAR = dehydroascorbate reductase, Gly = glyoxalase, GP = guaiacol peroxidase, GPX = glutathione peroxidase, GR = glutathione reductase, GSH = glutathione, GSSG = glutathione disulfide, GST = glutathione S-transferase, HSP = heat-shock protein, sHSP = small heat-shock protein, MAPK = mitogen-activated protein kinase, MT = metallothionein-like protein, RBOH = respiratory burst oxidase homologue, MDA = malondialdehyde, MHS = modified Holgan solution, POX = peroxidase.

Cross-acclimatization to various stresses is also a phenomenon that can be used in practice. It is based on sustained transcriptional memory lasting several days, weeks or a few progenies.^127^ This can be particularly useful if cross-acclimation can be triggered for an agriculturally significant trait, as examples listed in Table 1.

### 6.3. Memory modification intra- and interspecifically by sRNAs

sRNAs may also serve as a tool in communication between plant–pathogen (fungal, bacterial, etc.) and plant–plant interactions (plant–plant interactions include parasitic plants and their host). The mechanism is known as cross-kingdom RNAi, which is also bidirectional. Suppose a plant is attacked by a pathogen, the pathogen sends sRNAs to the host plant, resulting in the silencing of the immune response. In turn to this process, the host plant cells also deliver sRNAs by their extracellular vesicles to the pathogen. As a result, genes associated with virulence are silenced. The latter process is called host-induced gene silencing (HIGS). Providing efficient disease resistance by directly applying specific miRNAs, therefore circumventing transgenic strategies may be beneficial from an agricultural point of view.^128–130^

### 6.4. Pavlov’s rose? – associative use of the memory in plants and its practical importance

Above all, it is important to define precisely what can be considered as associative learning based on psychological and biological behavioral definitions^30^ and to group and interpret plant behavioral responses, accordingly. Habituation (i.e. the reduction or cessation of the response to a repeated stimulus), including also the generalization, and sensitization (i.e. the increase in the response to a stimulus) can be classified as non-associative learning. However, if a stimulus (CS; conditioned stimulus) is paired with another stimulus (UCS; unconditioned stimulus), and after the organism has been repeatedly exposed to this stimulus pair, the response can be elicited by the paired unconditioned stimulus (UCS) alone, then this behavior change is already an associative learning. In that case, UCS will be used as an indicator for CS, like in the case of Pavlov’s dog.^131^ The question was whether there is a Pavlov’s rose, that is, whether plants are capable of associative learning.

In the last decade, more and more evidence has emerged regarding the ability of plants to form associations and use their memory associatively.^132,133^ Experimenting with peas (*Pisum sativum* L. cv. Massey Gem), Gagliano et al.^133^ demonstrated that the pea plants grown in a Y maze were able to develop an association and use their acquired information associatively. Growth toward the light, that is a biologically important stimulus in plants (blue light, in the role of UCS, the indicator), was thereby allowed under the influence of an associated neutral cue (airflow, in the role of CS), which predicted where the light will occur, even in spite of the prevailing light tropism in the plant. It has been proven that plants were able to perceive the stimulus (light) both in space and time and to build both positive and negative associations. Although Markel^134^ attempted to refute Gagliano et al.^133^ results in a repeated experiment, Gagliano et al.^135^ responded by pointing out that Markel’s different experimental setup was unsuitable for reproducing their experimental results. This reply of Gagliano, however, was further disputed by Markel,^136^ who pointed out that the lack of replicability cannot be explained by the differences in the experimental setups. Nonetheless, this was an attempt rather on reproducibility than on replicability considering the variations both in the materials (different genotypes, different plant age) and methods (f.e. lighting, soil, etc.).^137^ These material and method differences can cause different physiological traits and thereby different sensitiveness, responsiveness and different memory states in the experimental plants of both experiments.^138^

Bhandawat et al.’s^139^ study was the first and the only one to date with transcriptomic evidences for the associative use of the memory in plants. In their study, sound (green music, 50 dB) was the indicator (UCS) that associatively evoked the response to abiotic stress, the heat (CS) in *Arabidopsis*. Expression intensities of five genes (*HSFA3, ATHSSP101, ATCTL1, SMXL7*, and *CHIP*) that are known to be involved in the high temperature and heat shock response, were studied. Up-regulation of all five heat-responsive genes in varying degrees, but significantly, was detected in conditioned plants after music but without heat stress. Up-regulation of those genes was significantly higher than in plants treated only with music (non-conditioned). *HSFA3* and *ATCTL1* heat responsive genes seemed to be the better candidates for indicating associative learning in *Arabidopsis* plant.

We know that associative learning itself does not require consciousness or a nervous system.^140^ Although it may be possible even for unicellular organisms based on biochemical and genetic networks,^141^ this ability of plants is still disputed, even though it cannot be ruled out on a theoretical basis.^138^ The settling the debate is also not helped by the fact that there are currently very few targeted experiments regarding the associative learning of plants. The possibility, however, that plants can use their memory associatively, just like animals, can open up exciting perspectives. In the near future, we must prepare to grow crops that can efficiently and quickly adapt to the rapidly changing environment under the conditions of climate change. In light of this, the ability of plants to use their memory in an associative way is appreciated and it can be used to increase crop security, although tremendous experimental efforts and further evidences are still needed before it can be applied.

## 7. Conclusions

Plant memory is of acclimation, adaptation and evolutionary importance and based on the interplay of biochemical and molecular networks.^15,36,104,142^ Non-coding RNAs act on and regulate the different layers of the plant memory. RNA interference mechanisms act at the level of RNA and DNA and in their interaction,^71,72,90^ creating a link between the chromatin modification, transcriptomic and post-transcriptional modification layers of vegetal memory.

Using plant transcriptional memory implies the development of new breeding and cultivation methods, since it allows elaborating promising cost-efficient and effective strategies to cope with the ever-changing environmental perturbations, caused by climate change. Among possible, memory-based methods, the priming and cross-acclimation^36^ have been already used in practice as well. The global food production requires increase day to day, and the agricultural sector needs to satisfy these needs by producing plants that are well adapted to the changing environment and ensure adequate yield and quality. That is the main reason why the ‘Climate Smart Agriculture’ (CSA) is gaining ground nowadays. The biological product of CSA is climate-smart crops that should tolerate reoccurring stress events in the current and also in the future generations as well.^143,144^ Associative learning in plants is theoretically possible.^138–140^ Even if there is still limited experimental evidence for it,^3,133,139^ based on the limited results, it cannot be ruled out that in the near future it may provide a new opportunity for developing new cultivation strategies that allow rapid adaptation to a rapidly changing environment.

## Glossary

Acclimation: after exposure to non-life-threatening stress, plants change their response intensity
Hardening/sensitization: response intensity increases
Familiarization/habituation: response intensity decreases (but not with motor fatigue or sensory adaptation)
Training/conditioning: response intensity change depends on stimuli
Cis memory: If the memorization process involves physical marks on the chromatin (e.g.: DNA methylation, histone modifications).
DNA methylation/demethylation: reversible transfer of a methyl group to adenine, or cytosine. Role in the regulation of gene expression.
**Histone modifications (HPTMs)**: post-transcriptional modifications of the N-terminal tails of histones (e.g.: acetylation, phosphorylation, methylation, ubiquitylation, etc.)
smallRNAs/lncRNAs: regulation at transcriptomic and post-transcriptomic levels, small and long non-coding RNA sequences
**Cross-acclimation**: priming with a non-lethal stimulus result in stress tolerance to another, different stimulus.
Epialleles: variations in gene expression patterns arising from epigenetic modifications that represent different epigenetic states of a specific gene or genomic region. The presence of epialleles result in distinct phenotypic diversity
Epigenetics: is the study of heritable changes of gene expression patterns that does not include DNA sequence alterations. A form of reversible gene inheritance without changing the DNA sequence. Plant stress memory is mainly epigenetic based.
**Intergenerational memory**: when plant stress memory extends to the next stress-free generation.
**Intragenerational memory**: affects the individual plant, occurring during the span of one generation.
**Short-term memory**: can be reactivated within a limited period of time (usually about some days to few weeks).
**Long-term memory**: can be sustained long term across multiple developmental stages within the current life cycle.
**Meiotic memory**: subsists through meiotic cell divisions.
**Mitotic memory**: subsists through clonal reproduction, mitotic cell divisions.
Plant stress memory: the ability to store the changes after stress exposure and using the gathered information to better adapt to the changing biotic and abiotic environmental stress.
Priming: enhanced resistance for both abiotic and biotic stress after pre-exposure to those stresses/eliciting factors (synonym: stress imprint). During priming. a singular or multiple stress events prepares the plant to possible future stress, thereby improving their fitness.
Reset: Memory formation often needs trade-offs, regardless of the adaptiveness. When memories turn out to be maladaptive, it is advisable to consider resetting. Deletion of memories.
**Somatic memory**: lasts maximum for the lifetime of the individual plant, but in most cases, it is sustained only for a fraction of the lifespan.
**Transcriptional memory**: lasting changes in the gene expression levels after non-lethal stress and recovery, altered transcriptional responses. Mainly regulated by epigenetics. After an exogenous cue, relatively to the naïve state the changed gene expression patterns are sustained.
**Transgenerational memory**: subsists through the next two or more generations without stressors. The primed state transmissions from parental plants to the progeny.
**Trans memory**: if the memory is maintained through the concentration of a diffusible signal, transmitted by the cytosol partitioning, or by feedback loops.
